# In situ monitoring of the influence of water on DNA radiation damage by near-ambient pressure X-ray photoelectron spectroscopy

**DOI:** 10.1038/s42004-021-00487-1

**Published:** 2021-04-09

**Authors:** Marc Benjamin Hahn, Paul M. Dietrich, Jörg Radnik

**Affiliations:** 1 Institut für Experimentalphysik, Freie Universität Berlin, Berlin, Germany; 2 Bundesanstalt für Materialforschung und -prüfung, Berlin, Germany; 3 SPECS Surface Nano Analysis GmbH, Berlin, Germany

**Keywords:** Biophysical chemistry, DNA

## Abstract

Ionizing radiation damage to DNA plays a fundamental role in cancer therapy. X-ray photoelectron-spectroscopy (XPS) allows simultaneous irradiation and damage monitoring. Although water radiolysis is essential for radiation damage, all previous XPS studies were performed in vacuum. Here we present near-ambient-pressure XPS experiments to directly measure DNA damage under water atmosphere. They permit in-situ monitoring of the effects of radicals on fully hydrated double-stranded DNA. The results allow us to distinguish direct damage, by photons and secondary low-energy electrons (LEE), from damage by hydroxyl radicals or hydration induced modifications of damage pathways. The exposure of dry DNA to x-rays leads to strand-breaks at the sugar-phosphate backbone, while deoxyribose and nucleobases are less affected. In contrast, a strong increase of DNA damage is observed in water, where OH-radicals are produced. In consequence, base damage and base release become predominant, even though the number of strand-breaks increases further.

## Abstract (web summary)

Ionizing radiation damage to DNA plays a fundamental role in cancer therapy, whereby water radiolysis effects are important to take into account. Here, near-ambient-pressure X-ray photoelectron-spectroscopy is used to directly measure DNA damage during irradiation in water and compared to that in a dry atmosphere.

## Introduction

The damage to biomolecules caused by ionizing radiation is the reason behind treating of cancer via radiation therapy^1^. Hereby, DNA damage is of key interest due to its central role in reproduction and mutation. In isolated DNA molecules, the damage can occur at its different building blocks, the sugar–phosphate backbone, and the nucleobases. The most important types of damage, which can lead to genetic instability, are single strand breaks (SSB) and double strand breaks (DSB) at the sugar–phosphate backbone and the loss or chemical modifications of the nucleobases. To improve therapeutic outcome and to develop more effective radiosensitizers, a better understanding of the underlying damage mechanisms is necessary^1–3^. Due to the high amount of water in biological tissue, most of the inelastic scattering processes between the incoming high-energy radiation (*γ*) and tissue occur with the solvent. Photons used in radiation therapy have energies in the MeV range. At these energies, secondary particles are produced by inelastic scattering processes such as the photo-electric effect, Compton scattering, Auger effect, or pair production^4^. The former scattering events produce additional electrons, while pair production results in the creation of an electron–positron pair. For x-ray photons with 1.4 keV kinetic energy, as used in this study, ionization is the dominant inelastic scattering process^4^. The ionization of water molecules produces secondary particles as described by the net-ionization reaction^5,6^

1 $$\gamma +2\ {\mathrm{H}}_{2}\mathrm{O}\to {\mathrm{H}}_{2}{\mathrm{O}}^{+}+{e}^{-}+{\mathrm{H}}_{2}\mathrm{O}\to {\mathrm{H}}_{3}{\mathrm{O}}^{+}+\mathrm{O}{\mathrm{H}}^{\bullet }+{\mathrm{e}}^{-}$$

The secondary species produced by water radiolysis are most notably the hydroxyl radicals, ions, and secondary electrons^7–12^. These reactive species are produced in high amounts (above 10^4^ e^−^/MeV deposited energy) by the primary radiation^13,14^. Due to their high abundance, most of the DNA damage is attributed to these secondary species. Thereby, the hydroxyl radicals^12^, the low-energy electrons (LEE)^9^. and their successors, the prehydrated electrons^8,15–17^, are the most lethal agents. The radiation damage is categorized into direct effects, e.g., from ionization or excitation of biomolecules or by indirect effects, i.e., chemical modifications induced by reactive oxygen species (ROS)^7,9,12^. Despite many years of research, the relation of the damage from direct and indirect effects is still controversially debated^1,8,11,12,16–18^. This is owed to the experimental difficulties of simultaneously accessing both types of effects within the same experimental setup. Since the ROS are produced by water radiolysis (Eq. (1)), the indirect effects caused by ROS can only be observed in fully hydrated environment. On the other hand, most of the damage originating from direct effects is caused by secondary electrons with energies below 100 eV. Here, it is noteworthy that even LEE with energies below the ionization threshold can efficiently damage DNA by formation of transient negative ions (TNI) and subsequent dissociative electron attachment (DEA)^9,11,18^. The inelastic mean free path of these LEE at ambient pressure is in the order of nanometers. Thus, the investigation of their isolated effects on DNA was performed mostly in ultrahigh vacuum (UHV), with additional postirradiation analysis by biochemical methods, such as agarose gel electrophoresis or high-performance liquid chromatography^9,19^. To monitor radiation induced chemical transformation of functional groups in situ, without postirradiation treatment, x-ray photoelectron spectroscopy (XPS) is a versatile tool. During XPS measurements, the primary x-ray photons do not only produce secondary electrons, which interact with tissue and damage DNA, but they also produce photoelectrons which probe the chemical environment. So far, all previous work applying XPS to investigate radiation induced DNA damage was performed under UHV conditions, focusing on direct damage^19–25^. One of the first studies was performed by Ptasinska et al.^19^. There, predominantly, formation of strand breaks was observed. Furthermore, Vilar et al.^20^ studied the electron interaction with self-assembled DNA monolayers in the presence of Na^+^. They concluded that electrons interact mainly with the backbone. Rosenberg et al.^21^ focused on the relationship between interfacial bonding and radiation damage in double-stranded DNA (dsDNA) absorbed on gold surfaces. Xiao et al.^22^^,^^23^ investigated the enhancement of bond breakage in DNA by cisplatin radiosensitizers. The observed enhancement of bond breakage in DNA by cisplatin was attributed to the sensitization of DNA to LEE and an increased production of LEE at the site of binding of the radiosensitizer. Furthermore, McKee et al.^24^ used x-rays to eject LEE from a gold substrate and to probe the resulting damage in model systems of condensed nucleotides. This method was further developed by Kundu et al.^25^ by incorporating a separate LEE source to monitor the damage to deoxyadenosine monophosphate via XPS. All these studies focused on direct damage by x-ray photons and LEE since they were exclusively performed under UHV conditions. Nowadays, novel ambient pressure instrumentation allows such investigations in the presence of water. Here, we perform irradiation and simultaneous XPS measurements on fully hydrated DNA. This enables the study of changes of damage yields caused by the presence of H_2_O, via different mechanisms, such as the production of ROS, modification of electron transfer (ET) channels, or conformational changes of DNA.

## Results

**Fig. 1:**
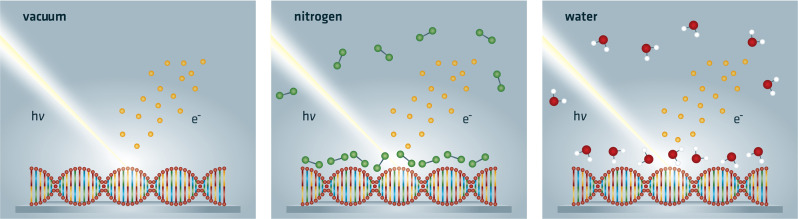
Sketch of the three irradiation conditions applied. X-rays pass through the vacuum, nitrogen, or water atmosphere, scatter at the DNA, and eject photoelectrons.

Here, we present for the first time simultaneous induction and probing of ionizing radiation damage to DNA by near-ambient pressure (NAP) XPS under H_2_O and N_2_ atmospheres, as well as standard UHV conditions (Fig. 1).

### Direct DNA damage

**Fig. 2:**
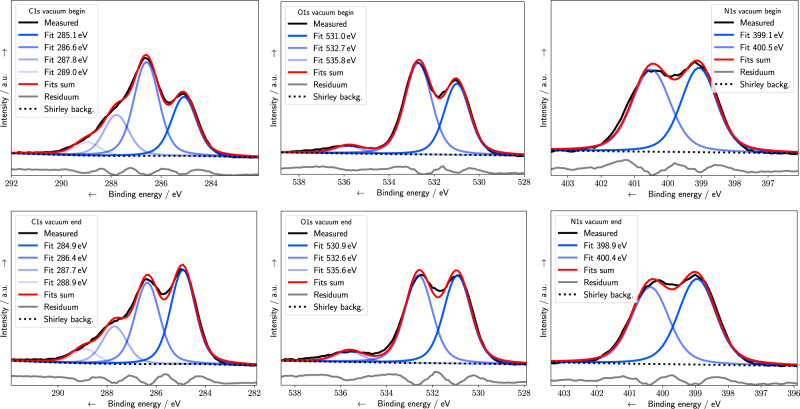
XPS spectra under vacuum conditions. C1s (left column), O1s (central column), and N1s (right column) spectra are shown at the beginning (top row) and end (bottom row) of the exposure. In addition, Voigt peak fits (blue), the sum (red), the Shirley background (black dotted curve), and fitting residuals (gray) are also shown. Residuals were shifted to lower *y* values.

**Fig. 3:**
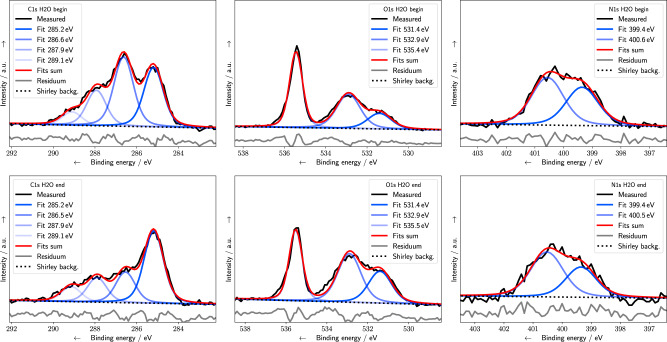
XPS spectra under water atmosphere. C1s (left column), O1s (central column), and N1s (right column) spectra are shown at the beginning (top row) and end (bottom row) of the exposure. In addition, Voigt peak fits (blue), the sum (red), the Shirley background (black dotted curve), and fitting residuals (gray) are also shown. Residuals were shifted to lower *y* values.

**Fig. 4:**
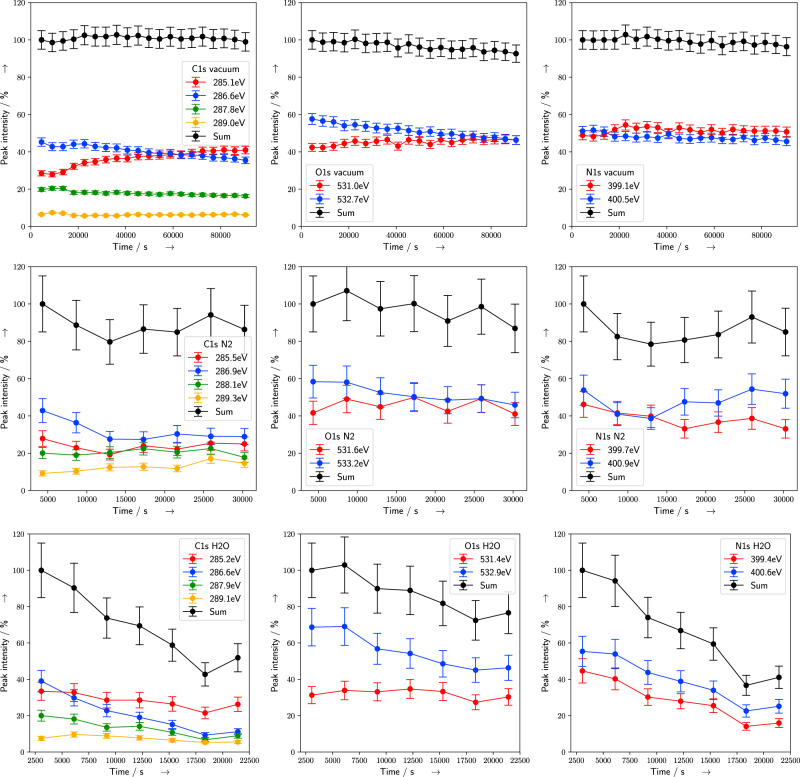
Time dependent normalized peak areas of XPS signals. Evolution of XPS signals at C1s (left column), O1s (center column), and N1s (right column) binding energies are shown for vacuum (top row), N_2_ (center row), and H_2_O (bottom row) conditions. Intensities were normalized on the P2p signal (compare SI, Fig. S3) at the same time. The percentage of the intensity is given with respect to the total integrated peak area at the beginning of the irradiation. Assignments are according to Table 1. Error bars were determined using a matrix inversion approach (compare SI, Sec. 1.4). Lines are guides to the eye.

In the following, the vacuum results are compared with previous studies, before the modifications of the underlying damaging channels by the presence of N_2_ and H_2_O atmosphere, are discussed. In Fig. 2, photoelectrons ejected under vacuum conditions at C1s (left column), O1s (central column), and N1s (right column) binding energies (BE) are shown together with the deconvolution results at the beginning (top row) and end (bottom row) of the exposure. The peaks are assigned to different chemical bonds (Table 1) based on literature values^19,21,26^. The C1s signal is deconvoluted into four different peaks at BEs at (1) 285 eV which belongs to hydrocarbons, (2) around 286–287 eV originating from alcohol (C–OH), backbone (C–O–P), cyclic ether (C–O–C), and carbon bond to nitrogen (C–N), while (3) at 288 eV to C=O, C=N, and (4) at 289 eV to N–(C=O)–N. The O1s spectra are deconvoluted into components assigned either to double bonded oxygen at 531 eV (C=O, P=O) or single bonded oxygen in the backbone (C–O–P), sugar (C–O–C), or alcohol (C–OH) at 532 eV, as well as water at a BE of around 536 eV. The N1s spectra are deconvoluted into contributions from imines around 399 eV and from amines, amides, and urethanes around 400.5 eV. Alternative deconvolution strategies based on three peaks are discussed in the Supplementary Information (SI), Sec. 1.4. Changes in the peak intensities during the course of the irradiation (Figs. 2 and 3) occur due to damage at the different DNA subunits. An increase of a certain species can be assigned to an addition to or formation of radicals at the DNA, which are precursors of new products. Decrease of a relative peak area with time corresponds to the cleavage of an associated bond (Table 1) or the release of a fragment from the surface^19^. The various types of DNA damage, namely strand breaks, sugar decomposition, and base damage, are related to different molecular groups. Although additional minor effects might contribute to the chemical changes observed, they are still of highest biological relevance, since they can be the starting point of mutation and apoptosis^7^. Thus, changes of the N1s signals can be assigned to dehydrogenation of the amino groups or to the breaking of the N-glycosidic bond, which can lead to a base release^19^. Sugar decomposition can result in the loss of C–OH groups and a decrease of the C1s and O1s signals over time. The decrease of signals associated with the C–O–P bonds at the DNA backbone at C1s 286 eV and O1s 532 eV with simultaneous formation of P–O^−^ and C–C^•^ at 285 eV can be interpreted as formation of strand breaks at the sugar–phosphate backbone^21^. This behavior, associated with strand break induction, is the predominant trend observed during XPS measurements under vacuum conditions (Fig. 4, top row). On the same time scale, the total peak intensities and the peak areas associated with nitrogen bonds are relatively unaffected. Both trends are in excellent agreement with XPS data of dsDNA as measured by Rosenberg et al.^21^. Furthermore, the slight decrease of the N1s signal at 400.5 eV compared to the imine signal (Fig. 4, top row right) is similar to the results by Ptasinska et al. reported for calf-thymus dsDNA^19^. Recent results from XPS studies on nucleotides show similar trends of oxygen-related peaks, while differing for the imine-related signals which decreased during irradiation^24^. This variation might be explained by the differences in LEE localization at the nucleobases between dsDNA and single nucleotides. Especially, since the formation of a TNI at the nucleobases and subsequent ET to the backbone is one of the most frequent damage pathways for strand break induction by LEE^11,27^. All this damage can be attributed to direct effects originating from x-ray photons or secondary electrons, since water is mostly absent under vacuum conditions. Here, on average less than 0.3 water molecules per nucleotide were present, as determined from the O1s spectral intensities (Fig. 2, center column). Thus, the initial distribution of direct ionization events at DNA molecules correlates with the electron density at the different subgroups^7,28^. Upon ionization, an electron is ejected, while the hole can migrate within the DNA. The hole can lead to a SSB via reactions at the DNA backbone or localize at the bases with a preference for guanine^28,29^. Besides ionization events, LEE with energies below 20 eV can form TNI and damage DNA by resonant processes such as DEA or shape resonances. The associated LEE damage yields depend on the initial electron capture probability of the DNA bases to form TNIs. Strand breaks are predominantly produced by cleavage of the C–O bond in the backbone, while base releases occur after cleavage of the N-glycosidic bond^30^. For LEE between 4 and 16 eV, the yield of strand breaks is approximately twice as high as the yield of base release^31^. Thus, taking into account the high abundance of LEE and their preferential cleavage of the DNA backbone (Fig. 2), we can confirm previous studies^21,24,25^, and conclude that LEE cause the majority of the strand breaks observed under vacuum conditions.

**Table 1:** Peak assignment and binding energies.

Name	BE/eV	Bonds	Groups
C1s	285	C–C, C–H	All
C1s	286–287	C–O, C–N	All
C1s	288	C=O, C=N, C–NH_2_	Bases
C1s	289	N–(C=O)–N	Bases
N1s	398–400	N=C, –N=	Bases
N1s	400–402	NC_3_, –NH_2_, O=C–N–C=O	Bases
N1s	405–406	N_2_	Nitrogen
O1s	531	C=O, P=O	Bases, backbone
O1s	532–533	C–O–C, C–O–P, C–OH	Sugar, backbone
O1s	534–536	H_2_O	Water
P2p	133	P2p_3/2_	Backbone
P2p	134	P2p_1/2_	Backbone

Binding energies (BE) and their assignments are based on the literature as referenced throughout the text. All BE were referenced to the Fermi level.

### The influence of nitrogen

The measurements we presented so far were performed in an UHV-XPS system which provides the charge compensation needed in vacuum. They were used to compare the behavior of our DNA samples with the literature data. In contrast, during NAP-XPS measurements, the charge compensation is achieved by the presence of gases. Since vacuum and NAP measurements were performed in devices which differ in a variety of properties (e.g., x-ray fluence, incident angle, spot geometry, transmission function, charge compensation), results can only be compared qualitatively. Thus, to enable a direct comparison between hydrated and nonhydrated DNA, measurements under N_2_ and H_2_O atmosphere were performed within the same NAP-XPS setup (compare SI, Fig. S2). The qualitative evolution of the XPS data between vacuum (Fig. 4, top row) and N_2_ (Fig. 4, center row) are similar, even though the overall damage observed over time is slightly higher for N_2_. Here, modifications of various damaging channel under the presence of N_2_ might occur. Thus, x-ray interactions with N_2_ molecules have to be considered, since their dissociation products are also potential damaging agents. However, in a previous study it was concluded that irradiated pure N_2_ produces much less damage by indirect effects than other gases, such as O_2_ or NO_2_, which agrees with our results^32^. There, it appeared that N_2_ molecules mostly damage DNA in combination with ROS, which are not present under the anaerobic conditions applied here. Furthermore, at NAP conditions, the gas molecules have a mean free path of less than 1 μm. Thus, only x-ray interaction near the surface can produce reactive species which are able to reach the DNA^32^. To gain deeper insight into the inelastic scattering processes, and herewith to direct damage effects, the production of reactive species and the energy deposit in DNA, particle scattering simulations were performed. Hereby, the impact of x-ray photons passing through the respective vacuum, nitrogen, or water atmosphere, an additional surface layer of absorbed gases, and the DNA itself was simulated. Since XPS approximately probes the DNA until a depth of 10 nm, only scattering events in this region were evaluated. The results show that each photon deposits on average 1.9 eV in the DNA layer, whereby less than 0.2 % of them ionize a DNA molecule and produce an electron–hole pair. Approximately, the same number of electrons thermalizes in the DNA region, and therefore are able to form TNIs. All these results vary less than 5% between vacuum, N_2_, and H_2_O atmosphere (compare SI, Table S3). Thus, the changes observed in DNA damage induction under different atmospheric conditions (Fig. 4) are not caused by direct effects. From these considerations, we can conclude that experiments under N_2_ atmosphere allow us to study direct damage effects caused by high-energy radiation and LEE, as suggested by Alizadeh et al.^32^. Thus, comparison with results from water atmosphere allows in situ monitoring of chemical changes induced by indirect effects.

### DNA damage in water

Essentially, the overall damage increases strongly under water atmosphere. The decrease of the total C1s, O1s, and N1s peaks relative to the P2p signal (compare SI, Fig. S3) indicates a desorption of damaged, volatile subgroups (Fig. 4, bottom row). The behavior differs in terms of quality and quantity substantially from irradiation under N_2_ atmosphere. Hereby, the most striking damage increase can be attributed to peaks associated with nitrogen bonds (see Table 1 and the simultaneous decrease of the normalized C1s and N1s peaks in Fig. 4, bottom row left and bottom row center) which are located exclusively in the nucleobases. It must be noted that the loss of nitrogen is correlated with the decrease of the C1s peak at 286.6 eV, whereas the feature at 285.2 eV is nearly constant. The other peaks at 287.9 and 289.1 eV show only a slight decrease which is accompanied by the decrease of the O1s. Thus, the decrease of the carbon can be explained by the loss of the nitrogen and carbon containing components, the nucleobases. Especially, base release is likely, since the nucleobases are only bound to the DNA backbone by the N-glycosidic bond (C1s at 286 eV and N1s at 400 eV). Furthermore, strand break induction, which was the dominant type of damage under vacuum and N_2_ conditions, increased further when water was present. Ionization of water molecules leads to the formation of hydroxyl radicals (Eq. (1)), which cause damage classified as indirect. The ^•^OH-radicals react selectively with the electron richer regions of DNA. Addition to C=C and C=N double bonds in the purines is performed at nearly diffusion-controlled rates. This explains the decrease of the C1s 288 eV peak, which is only observed under water atmosphere (Fig. 4, bottom row). Since C=O bonds are electron deficient at the carbon atom, additions are uncommon here. Thus, the decrease of the C1s 288 eV signal is mostly due to base damage. Hydroxyl radical induced H abstraction or damage via ET is possible, but less likely^12^. The spectral signature of these processes is the decrease of C–H signals (C1s 285 eV), while formation of related C–O bonds stands in direct competition with decrease due to simultaneously occurring degradation processes. Hereby, H abstraction happens at the deoxyribose and can lead to abasic sites, alkali labile sites, or strand breaks via multiple reaction steps. In addition, ionization of water molecules in the direct vicinity of DNA can lead to a radical transfer from H_2_O^+^ to DNA, producing a hole, as discussed above^7^. The other radical species of importance H^•^ and the (pre)solvated electron behave in many ways similarly and damage biomolecules by ET induced reactions. In addition, H^•^ can perform addition to C=C double bonds with preferences for electron rich sites. But the rates are generally much lower than for OH-radicals, thus no absolute increase of the C1s 285 eV signal is expected. Furthermore, the formation of radical sites at the sugar can lead to a reaction with surrounding water molecules and result in a base release^12^. Therefore, the presence of water not only causes indirect damage, but also modifies the outcome of multiple step reactions involving radical sites, regardless of their origin^33^. Such modifications can be caused, for example, by formation of new hydrogen bonds with water molecules and result in conformational changes of the DNA molecule^34^. When electrons lose their kinetic energy by inelastic scattering at water molecules, they can get trapped in a prehydrated state, before becoming fully hydrated^6,35^. Currently, the role of these prehydrated electrons, their exact ET mechanisms to DNA and damage mechanisms are disputed^8,15–17,36,37^. Here, NAP-XPS in combination with a synchrotron light source, tuned to excite electrons directly into the energy band at the DNA-water interface, may provide valuable information about the damage processes triggered by ET from the prehydrated state. Finally, the reactivity of fully solvated electrons is much lower compared to the aforementioned processes, and strand break induction was not observed so far. Therefore, their damage contribution is negligible under the given conditions.

## Discussion

**Fig. 5:**
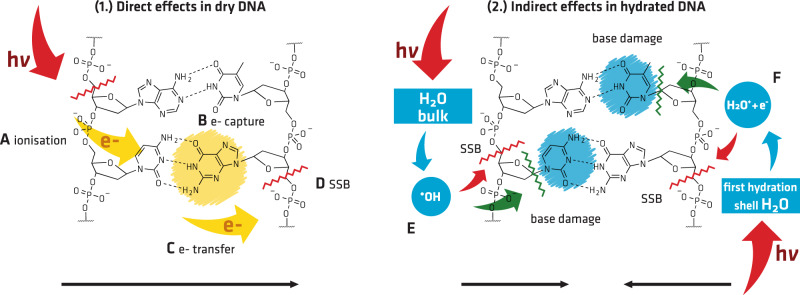
Overview of the most important (1.) direct and (2.) indirect damage mechanisms. In dry DNA (1.) direct effects produce mostly SSB (red lines) via ionization events (**A**) of DNA (red arrows) or by LEE via the formation of TNI at the bases (**B**), subsequent transfer to the backbone (**C**), and rupture of C–O bond (**D**). In contrast, a shift toward base damage is observed in hydrated DNA (2.). Here, indirect damage channels contribute to the increase of the overall radiation damage. Ionization of water molecules in the bulk (**E**) or in the first hydration (**F**) layer represents the most important processes. Direct processes are present in hydrated DNA, but are not shown for simplicity. Green lines represent base loss. For details see the text.

Upon hydration of DNA, an overall increase of the damage was found. The strand break induction, as determined by the relative change of the C1s signal at 286 eV, was approximately twice as high in hydrated DNA (Fig. 4, left column bottom row) as in DNA under nitrogen atmosphere (Fig. 4, left column center row). The base damage was estimated by the decrease of the total nitrogen signal during the irradiation. In hydrated DNA (Fig. 4, right column bottom row), the total nitrogen signal decreased about three time stronger than in DNA under nitrogen atmosphere (Fig. 4, right column center row). This increase in radiation damage can be expected due to the additional contributions from indirect damage channels^6,14,34^. For example, Alizadeh and Sanche studied DNA strand break yields of x-ray irradiated DNA films under different levels of hydration^38^. They found ~1.5–2 times increase of the SSB yield by comparing dry DNA with hydrated DNA, with one layer of hydration, while for DNA in bulk water a threefold enhancement of the SSB yield was observed. Thus, our results are in good agreement with their data for DNA with one hydration layer. In contrast, for base damage, Swarts et al. found little difference between dry DNA and DNA with only one hydration layer. There, only the contributions from bulk water were observed to be 3.3 times more effective in leading to a base release, than ionization events in the first hydration layer^34^. They concluded, that in an environment where bulk water is formed, base release is caused mostly by hydroxyl radicals (Fig. 5E), and only to a lesser extent by charge transfer from the first hydration layer (Fig. 5F). Under NAP conditions only 5–10 water molecules per nucleotide are present. To form a complete second hydration layer about 20 water molecules per nucleotide are necessary^6,34^. Thus, here, the relative amount of water is lower than in a cellular environment. Hence, compared to a cell, the present experiments can be expected to have a relative higher contribution from processes involving H_2_O^+^ than ^•^OH^34^. Therefore, the strong shift from the predominance of strand break induction under vacuum conditions toward base damage under water atmosphere is somewhat unexpected. These results suggest a selectivity for DNA base damage in dependence of DNA hydration. Some of the differences observed might be attributed to variation of DNA base sequence, hydration dependent inter- and intrastrand coupling, conformation, and the local environment between the various studies. Especially, the change of DNA conformation, due to the formation of additional hydrogen bonds with absorbed water molecules, has to be considered. In the dry state, DNA resides in the so called pseudo C-form. Upon hydration it changes into the native B-form^34^. However, according to the literature the pseudo C-form is known to have higher *G*-values from ionization events for base release than the B-form^34^. Thus, the observed increase of base damage is unlikely to originate from a change of conformation and a resulting higher effectiveness of direct effects. Therefore, it is attributed to the products of radiation interaction with water, including OH-radicals from the DNA surroundings (Fig. 5E) or short lived water cations located in the first hydration shell (Fig. 5F)^38^. A further source of variation of the damage yields is the difference in analytical methods applied. For example, throughout the literature SSB, DSB and base damage are mostly quantified by postirradiation analysis, where additional sample processing steps are involved^6,14,39^. There, only selected base damage can be accessed by targeted endonuclease assays^40,41^. Furthermore, mass spectrometry, which is often used to access base loss, only provides information about molecular groups which are able to escape from the surface^33,34^. In contrast, XPS has the advantage that it directly probes radiation induced chemical modifications in situ. Therefore, it is agnostic regarding the type of damage probed. This provides a substantial advantage compared to the damage specific methods applied in mechanistic studies of radiation induced DNA damage. To compare our results with data from radiobiological studies, we have to take into account that within the nucleus of mammalian cells counterions, histones and other cosolutes are present. They are known to alter the damage yield by shielding of DNA against OH-radical attack, or altering the damage efficiency of LEE^42–46^. Despite these differences in the local DNA environment, the results presented in this study are in good agreement with radiobiological studies^7^. For example, Ward estimated a base to sugar damage ratio of 2.7 for cellular DNA^7^. Thus, the experimental conditions of this study represent a valuable model system for the investigation of DNA damage on the molecular level. However, detailed NAP-XPS studies on the effects of an environment closer to the nucleus of mammalian cells will be performed in future work. Furthermore, NAP-XPS allows for flexible sample exposure to different gases. Therefore, it provides a unique opportunity to study oxygen induced fixation of radiation damage by applying aerobic and anaerobic conditions. In this context, the underlying chemistry of DNA–protein crosslinks formation can be studied in the future. These crosslinks are predominantly formed under low-oxygen concentrations and occur in cancerous tissue with high radiation resistance. Here, the combination of NAP-XPS with mass spectrometry will allow for detailed investigation of desorbed groups and provide complementary information^33^.

In summary, we have quantified radiation induced damage at fully hydrated DNA molecules directly by XPS. The comparison between irradiations under N_2_ and H_2_O atmosphere revealed a strong increase in the overall damage upon hydration. Contributions from direct and indirect processes were separated by combining NAP-XPS experiments with Monte Carlo particle scattering simulations. In dry DNA, high-energy photons and LEE showed a preference for the induction of DNA strand breaks at the backbone. In contrast, deoxyribose and nucleobases were affected much less by direct damage. This behavior changed dramatically when water was present. Here, excited water molecules and hydroxyl radicals initiated indirect damage processes which lead to base modification and base release. It was revealed that base modifications become predominant here, even when the total amount of strand breaks increased further as well.

## Methods

### Sample preparation

Herring sperm DNA in ultrapure water was obtained from Sigma-Aldrich. The DNA was dropcasted on carefully cleaned microscopy slides and dried with Ar gas (Linde). All three DNA samples were stored on dry ice until they were placed in the XPS chambers.

### XPS measurements

All XPS measurements were performed with Al K*α* radiation (*E* = 1486.6 eV). The UHV-XPS measurements were done with an AXIS Ultra DLD photoelectron spectrometer (Kratos Analytical, Manchester, UK). Here, the pressure was below 1 × 10^−8^ mbar. Laboratory NAP XPS measurements were done with an EnviroESCA (SPECS GmbH, Berlin, Germany)^47,48^. During the NAP-XPS measurements, the pressure was kept in the NAP regime between 4 and 14 mbar. Details on experimental procedures (SI Secs. 1.1 and 1.2) and data analysis (SI Sec. 1.3) are provided in the SI. XPS fitting results are summarized in SI in Tables S1–S3.

### Particle scattering simulations

Particle scattering simulations were performed with the Geant4 10.5 framework and the Topas 3.3 interface, the Livermore scattering cross sections and 1 nm particle cut length^49,50^. Simulation details are given in SI Sec. 2 and results are summarized in SI Table S4.

## Supplementary information

Supplementary Information

## Data Availability

The data that support the findings of this study are available from M.B.H. upon request.
