# Supplementary material for: In situ monitoring of the influence of water on DNA radiation damage by near-ambient pressure X-ray photoelectron spectroscopy
Source: Commun Chem. 2021 Apr 9;4:50. doi: 10.1038/s42004-021-00487-1 (PMC9814248; doi:10.1038/s42004-021-00487-1)
Supplement: Supplementary file 1 — Supplementary Information [file 42004_2021_487_MOESM1_ESM.pdf]

SUPPORTING INFORMATION: *In-situ* monitoring of the influence of water on DNA radiation damage by near-ambient pressure X-ray photoelectron-spectroscopy

Marc Benjamin Hahn[1,2,*], Paul M. Dietrich[3], and Jörg Radnik[2]

[1]Freie Universität Berlin, Institut für Experimentalphysik, 14195 Berlin, Germany
[*]hahn@physik.fu-berlin.de
[2]Bundesanstalt für Materialforschung und -prüfung, 12205 Berlin, Germany
[3]SPECS Surface Nano Analysis GmbH, 13355 Berlin, Germany

1 Supplementary Methods

1.1 Experimental details of vacuum XPS measurements

The UHV-XPS measurements were done with an AXIS Ultra DLD photoelectron spectrometer (Kratos Analytical, Manchester, UK) with monochromatic Al Kα radiation (E = 1486.6 eV). The pressure was below $1 \cdot 10^{-8}$ mbar. The electron emission angle was 0° and the source-to-analyzer angle was 60°. The binding energy scale of the instrument was calibrated following a Kratos analytical procedure which uses ISO 15472 binding energy data[3]. Spectra were taken by setting the instrument to the hybrid lens mode and the slot mode with a $300 \times 700\,\mu m^2$ analysis area. Furthermore, the charge neutralizer was also used. During the vacuum measurements the measurement of C1s, O1s, N1s, and P2p regions took approximately 4525 s.

1.2 Experimental details of NAP XPS measurements

Laboratory Near-Ambient Pressure X-ray Photoelectron Spectroscopy (NAP XPS) measurements were done with an EnviroESCA (SPECS GmbH, Berlin, Germany).[1, 2] The monochromatic Al Kα x-ray source is separated from the measurement chamber by a silicon nitride window, and the hemispherical energy analyzer is under ultra-high vacuum ($< 1\times10^{-8}$ mbar) due to a three stage

differential pumping system between the analysis section and analyzer. The entrance aperture (nozzle) has a diameter of 300 μm and the usual working distance is 1-2 times the nozzle diameter. With this set-up, it is possible to measure gaseous, liquid as well as solid samples at pressures up to 50 mbar. For ambient pressure measurements DNA samples were inserted into the EnviroESCA and the pressure was slowly reduced below 14 mbar to allow residually dissolved gases to evaporate. During the NAP-XPS measurements of about 8 hours, the pressure was kept in the near-ambient pressure regime between 4 mbar-14 mbar. All survey spectra were acquired with a pass energy of 100 eV, a step size of 1.0 eV, and a dwell time of 0.1 seconds. High-resolution core-level spectra (O1s, N1s, C1s, and P2p) were recorded in fixed analyzer transmission (FAT) mode at pass energy of 50 eV, a step size of 0.2 eV, and a dwell time of 0.1 seconds. The binding energy scale of the instrument was calibrated according to ISO 15472.[3] The electron emission angle was 0° and the source-to-analyzer angle was 55°.

1.3 Analysis of the XPS data

Deconvolution of the XPS signals was performed with the *Fityk* software and a Levenberg-Marquardt algorithm from the *MPFIT* library.[4] A Shirley background was subtracted from all spectra. The BE values of the spectra measured under UHV conditions were charge corrected on the C1s BE at 285 eV. Under NAP conditions, no further correction was needed since sufficient charge compensation is provided by the surrounding gas. Additionally, five subsequently measured spectra were averaged for each analysis due to the lower signal-to-noise ratio under NAP conditions. The time dependent evolution and comparisons of the C1s, O2s, and N1s regions are normalized on the total integrated peak area of the P2p region at the same time. This is legitimate since the amount of phosphate groups, which are only present in the DNA backbone, can be assumed to be constant during the irradiation. This is based on the fact that the DNA backbone is covalently bound twice to the rest of the molecule and complex damage is a rare event for the doses applied in this study.[5, 6] The percentage of the intensity in the time dependent figures is given with respect to the total integrated peak area at the beginning of the irradiation. Voigt peaks with fixed shape of 0.3 (Lorentz-Gauss ratio) were fitted to the spectra based on the assignments from the literature given and summarized in table 1 of the main text. The peaks were constrained to a range of $\pm$0.2 eV around their center positions within the same measurement series, either, UHV conditions, N_2 or H_2O atmosphere. Within each envelope the FWHM was constrained to $\pm$0.05 eV (compare Tab. S1-S3). One exception was made for the case of the O1s spectra under water atmosphere. There, the gas peak of water around 535 eV binding energy was fitted with an independent FWHM. We note here, that in the case of the O1s and N1s spectra, a deconvolution with three peaks is a viable alternative to reduce the fitting residuals to some extent (compare Fig. S1 right). Thereby, the imides at around 399 eV, the amides and

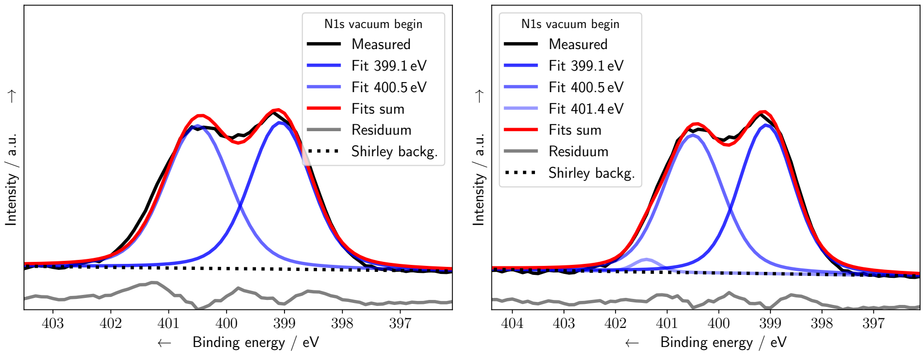

Figure S1: Comparison of two (left) and three (right) fitted peaks at the N1s data under vacuum conditions. For details see the text.

amines connected to the ring-structure of the nucleobases (around 400 eV) are complemented by a third N1s peak (around 401 eV) which is sometimes assigned either to hydrogen bonded or protonated amine species, N-C=O, or base-substrate interaction.[7, 8, 9] Nevertheless, to enable a comparison with literature values from the referenced damage studies, we have chosen to stick to a deconvolution of the N1s signal into to two Voigt peaks, as discussed in the main text. Since the argumentation related to N1s signal intensities in the main text is based on the evolution of the sum of the N1s under different conditions, a different peak deconvolution methodology would not alter the outcome here. The signal contribution from water in the O1s binding energy region was assigned, based on the work by Patel *et al.*[2] The relative uncertainties for peak area calculation under NAP conditions were estimated with a maximum value of 15 % using a matrix inversion approach.[10] Due to the better signal-to-noise ratio 5 % were achieved for the UHV measurements. An overview about spectra recorded under nitrogen atmosphere is given in Fig. S2. Peak fit parameters and results for all spectra at the beginning and end of the irradiations are summarized in Tab. S1-S3.

2 Particle Scattering Simulations

Particle scattering simulations were performed with the *Geant4* 10.5 framework and the *Topas* 3.3 interface. The *g4em-livermore* physics-list was enabled with a 1 nm particle cut length.[11, 12] During the simulation, multiple-scattering processes were disabled to increase the accuracy, and Auger, AugerCascade and Fluorescence calculations were enabled. 10^7 primary photons with 1.487 keV primary energy were simulated. For each experimental setting, *i.e.* vacuum, nitrogen or water atmosphere, an independent simulation was performed. The vacuum was modeled with the standard “vaccum” settings, as included in *Topas*, NAP conditions with a temperature of 300 K, a pressure of 2000 Pa and a den-

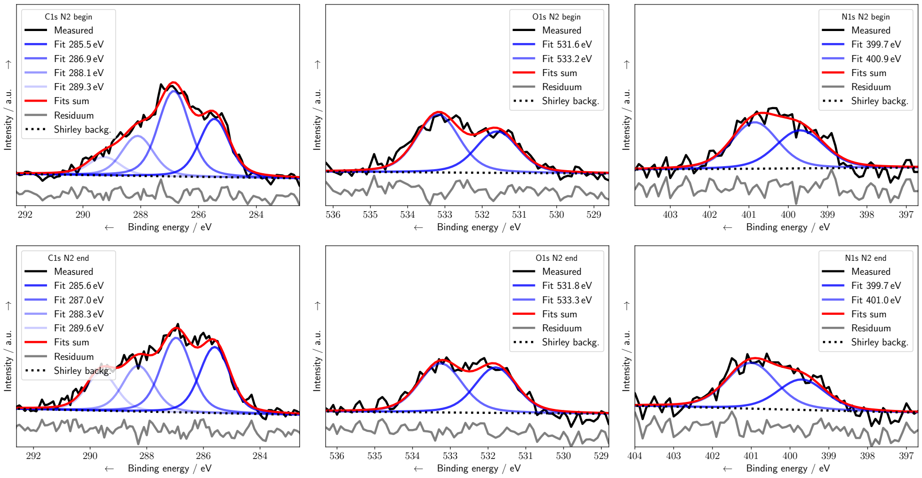

Figure S2: XPS spectra under nitrogen atmosphere. C1s, O1s and N1s spectra at the beginning and end of the exposure. Additionally Voigt peak fits (blue), the sum (red) and fitting residuals (grey) are shown. For better visibility, the residuum was shifted towards lower y values.

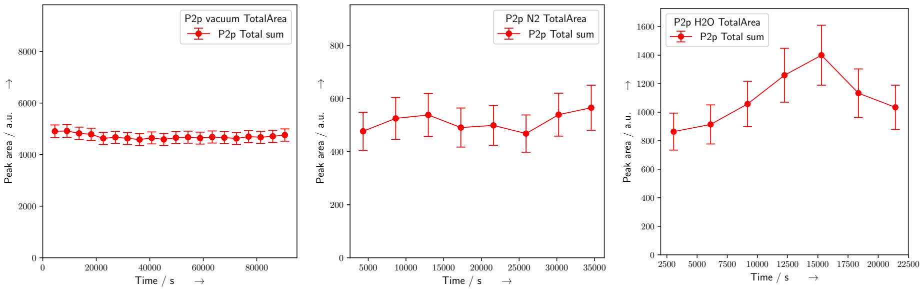

Figure S3: Time evolution of the total area of the P2p signal used for the normalization procedure of the C1s, N1s and O1s time evolution as given in the main text. Error bars were determined using a matrix inversion approach, for details see the text.

Table S1: Voigt peak fitting parameter and results for the XPS spectra of DNA in vacuum. All values were rounded to the first decimal point.

Spectra	Time	Center / eV	Area	FWHM / eV
C1s	Start	285.1	12393.6	1.2
C1s	Start	286.6	13927.3	1.2
C1s	Start	287.8	6073.8	1.2
C1s	Start	289.0	2119.6	1.2
O1s	Start	530.9	23121.4	1.3
O1s	Start	532.7	31430.1	1.4
O1s	Start	535.8	2568.4	1.4
N1s	Start	399.1	9837.7	1.3
N1s	Start	400.5	10281.5	1.3
C1s	End	284.9	14276.2	1.2
C1s	End	286.4	12354.5	1.2
C1s	End	287.7	5685.5	1.2
C1s	End	288.9	2168.9	1.2
O1s	End	530.9	24530.0	1.4
O1s	End	532.6	24499.6	1.4
O1s	End	535.6	3014.7	1.4
N1s	End	398.9	9906.6	1.3
N1s	End	400.4	8899.6	1.3

Table S2: Voigt peak fitting parameter and results for the XPS spectra of DNA in nitrogen. All values were rounded to the first decimal point.

Spectra	Time	Center / eV	Area	FWHM / eV
C1s	Start	285.5	310.9	1.2
C1s	Start	286.9	478.4	1.3
C1s	Start	288.1	224.1	1.3
C1s	Start	289.3	102.8	1.3
O1s	Start	531.6	388.3	1.3
O1s	Start	533.2	544.1	1.3
N1s	Start	399.7	224.1	1.4
N1s	Start	400.9	261.0	1.4
C1s	End	285.6	315.9	1.3
C1s	End	286.9	365.1	1.3
C1s	End	288.3	224.3	1.3
C1s	End	289.6	185.1	1.3
O1s	End	531.8	432.9	1.3
O1s	End	533.3	483.8	1.3
N1s	End	399.7	181.6	1.4
N1s	End	401.0	284.8	1.5

Table S3: Voigt peak fitting parameter and results for the XPS spectra of DNA in water. All values were rounded to the first decimal point.

Spectra	Time	Center / eV	Area	FWHM / eV
C1s	Start	285.2	833.7	1.1
C1s	Start	286.6	973.1	1.1
C1s	Start	287.9	498.3	1.1
C1s	Start	289.1	187.2	1.1
O1s	Start	531.4	1060.3	1.3
O1s	Start	532.9	2327.6	1.3
O1s	Start	535.4	2833.5	0.7
N1s	Start	399.4	499.2	1.3
N1s	Start	400.6	619.3	1.3
C1s	End	285.2	784.1	1.1
C1s	End	286.5	333.0	1.1
C1s	End	287.9	266.9	1.1
C1s	End	289.1	164.1	1.1
O1s	End	531.4	1228.6	1.3
O1s	End	532.9	1879.1	1.3
O1s	End	535.5	1541.7	0.8
N1s	End	399.4	213.4	1.2
N1s	End	400.5	336.8	1.3

sity of 22.5 μg/cm^3 for Nitrogen and a density of 14.4 μg/cm^3 for water, were used as a medium. The DNA was modeled with a density of 1.7 g/cm^3 with 50 % GC content and no salts present. The surface layer was assumed to have 1 nm thickness of either water or nitrogen and a density of 1 g/cm^3. Dose and energy deposit were scored within a surface area of 10^4 nm^2 and a depth of 10 nm for DNA. For the surface layer, the values were recorded for the depth of 1 nm and for the gas volume, within the last 99 nm before the sample. All values were determined by the dose, energy, charge and volume scorers as included in the *Topas* package. Results are summarized in Tab. S4.

Table S4: All values are given per 10^7 primary photons entering the area of $10^4\,nm^2$ within the first 10 nm of DNA, after a passage through vacuum, water or nitrogen gas and a layer of 1 nm absorbed gas molecules on top of the DNA. Note the distinction between ionisation events caused by x-ray photons (photoelectric effect) and electrons (electron-electron scattering). For details see the text.

Value	Vacuum	Water	Nitrogen
X-ray ionizations in $9.9 \cdot 10^5\,nm^3$ gas	1	5	3
X-ray ionizations in $10^4\,nm^3$ absorbed gas	-	1445	1148
Electron ionizations in $10^4\,nm^3$ absorbed gas	-	80	56
Netto e- flux in $10^4\,nm^3$ absorbed gas	-	1021	796
Energy deposit in $10^4\,nm^3$ absorbed gas	-	1.27 MeV	0.87 MeV
Dose in $10^4\,nm^3$ absorbed gas	-	5071 kGy	3490 kGy
X-ray ionizations in $10^5\,nm^3$ DNA	17698	17652	17644
Electron ionizations in $10^5\,nm^3$ DNA	7684	8009	7866
Total e- produced $10^5\,nm^3$ DNA	25382	25661	25510
Netto e- leaving the $10^5\,nm^3$ DNA volume	8299	7903	8027
Thermalized e- in the $10^5\,nm^3$ DNA volume	17083	17362	17483
Energy deposit in $10^5\,nm^3$ DNA	19.24 MeV	19.67 MeV	19.59 MeV
Dose in $10^5\,nm^3$ DNA	4533 kGy	4634 kGy	4616 kGy

Supplementary References

[1] M. Kjærvik, K. Schwibbert, P. Dietrich, A. Thissen, W. E. S. Unger, *Surface and Interface Analysis* **2018**, *50*, 996–1000.

[2] D. I. Patel, D. Shah, S. Bahr, P. Dietrich, M. Meyer, A. Thißen, M. R. Linford, *Surface Science Spectra* **2019**, *26*, 014026.

[3] ISO 15472:2010 Geneva, Switzerland. **2010**.

[4] M. Wojdyr, *Journal of Applied Crystallography* **2010**, *43*, 1126–1128.

[5] M. B. Hahn, S. Meyer, M.-A. Schröter, H. Seitz, H.-J. Kunte, T. Solomun, H. Sturm, *Physical Chemistry Chemical Physics* **2017**, *19*, 1798–1805.

[6] M. B. Hahn, S. Meyer, H.-J. Kunte, T. Solomun, H. Sturm, *Physical Review E* **2017**, *95*, 052419.

[7] N. Graf, E. Yegen, T. Gross, A. Lippitz, W. Weigel, S. Krakert, A. Terfort, W. E. S. Unger, *Surface Science* **2009**, *603*, 2849–2860.

[8] M. R. Vilar, A. M. Botelho do Rego, A. M. Ferraria, Y. Jugnet, C. Noguès, D. Peled, R. Naaman, *The Journal of Physical Chemistry B* **2008**, *112*, 6957–6964.

[9] M. Furukawa, T. Yamada, S. Katano, M. Kawai, H. Ogasawara, A. Nilsson, *Surface Science* **2007**, *601*, 5433–5440.

[10] R. Hesse, T. Chassé, P. Streubel, R. Szargan, *Surface and Interface Analysis* **2004**, *36*, 1373–1383.

[11] S. Agostinelli, J. Allison, K. Amako, J. Apostolakis, H. Araujo, P. Arce, M. Asai, D. Axen, S. Banerjee, G. Barrand, F. Behner, L. Bellagamba, J. Boudreau, L. Broglia, A. Brunengo, H. Burkhardt, S. Chauvie, J. Chuma, R. Chytracek, G. Cooperman, G. Cosmo, P. Degtyarenko, A. Dell'Acqua, G. Depaola, D. Dietrich, R. Enami, A. Feliciello, C. Ferguson, H. Fesefeldt, G. Folger, F. Foppiano, A. Forti, S. Garelli, S. Giani, R. Giannitrapani, D. Gibin, J. J. Gómez Cadenas, I. González, G. Gracia Abril, G. Greeniaus, W. Greiner, V. Grichine, A. Grossheim, S. Guatelli, P. Gumplinger, R. Hamatsu, K. Hashimoto, H. Hasui, A. Heikkinen, A. Howard, V. Ivanchenko, A. Johnson, F. W. Jones, J. Kallenbach, N. Kanaya, M. Kawabata, Y. Kawabata, M. Kawaguti, S. Kelner, P. Kent, A. Kimura, T. Kodama, R. Kokoulin, M. Kossov, H. Kurashige, E. Lamanna, T. Lampén, V. Lara, V. Lefebure, F. Lei, M. Liendl, W. Lockman, F. Longo, S. Magni, M. Maire, E. Medernach, K. Minamimoto, P. Mora de Freitas, Y. Morita, K. Murakami, M. Nagamatu, R. Nartallo, P. Nieminen, T. Nishimura, K. Ohtsubo, M. Okamura, S. O'Neale, Y. Oohata, K. Paech, J. Perl, A. Pfeiffer, M. G. Pia, F. Ranjard, A. Rybin, S. Sadilov, E. Di Salvo, G. Santin, T. Sasaki, N. Savvas, Y. Sawada, S. Scherer, S. Sei, V. Sirotenko, D. Smith, N. Starkov, H. Stoecker, J. Sulkimo, M. Takahata, S. Tanaka, E. Tcherniaev, E. Safai Tehrani, M. Tropeano, P. Truscott, H. Uno, L. Urban, P. Urban, M. Verderi, A. Walkden, W. Wander, H. Weber, J. P. Wellisch, T. Wenaus, D. C. Williams, D. Wright, T. Yamada, H. Yoshida, D. Zschiesche, *Nuclear Instruments and Methods in Physics Research Section A: Accelerators Spectrometers Detectors and Associated Equipment* **2003**, *506*, 250–303.

[12] J. Perl, J. Shin, J. Schumann, B. Faddegon, H. Paganetti, *Medical Physics* **2012**, *39*, 6818–6837.
